# High genetic and haplotype diversity in vaccine candidate *Pfceltos* but not *Pfrh5* among malaria-infected children in Ibadan, Nigeria

**DOI:** 10.7717/peerj.16519

**Published:** 2023-12-11

**Authors:** Mary Aigbiremo Oboh, Naemy Asmorom, Catherine Falade, Olusola Ojurongbe, Bolaji N. Thomas

**Affiliations:** 1 Biomedical Sciences, Rochester Institute of Technology, Rochester, NY, United States of America; 2 Pharmacology and Therapeutics, University of Ibadan, Ibadan, Oyo, Nigeria; 3 Medical Microbiology and Parasitology, Ladoke Akintola University of Technology, Ogbomosho, Osun, Nigeria; 4 Centre for Emerging and Re-emerging Infectious Diseases, Ladoke Akintola University of Technology, Ogbomosho, Oyo, Nigeria

**Keywords:** *Plasmodium falciparum*, Genetic diversity, Polymorphism, Phylogenetic relatedness, Sub-sahara Africa, Pfceltos, Pfrh5

## Abstract

Malaria remains a global public health challenge. The disease has a great impact in sub-Saharan Africa among children under five years of age and pregnant women. Malaria control programs targeting the parasite and mosquitoes vectors with combinational therapy and insecticide-treated bednets are becoming obsolete due to the phenomenon of resistance, which is a challenge for reducing morbidity and mortality. Malaria vaccines would be effective alternative to the problem of parasite and insecticide resistance, but focal reports of polymorphisms in malaria candidate antigens have made it difficult to design an effective malaria vaccine. Therefore, studies geared towards elucidating the polymorphic pattern and how genes targeted for vaccine design evolve are imperative. We have carried out molecular and genetic analysis of two genes encoding vaccine candidates—the *Plasmodium falciparum* cell traversal ookinetes and sporozoites (*Pfceltos*) and *P. falciparum* reticulocyte binding protein 5 (*Pfrh5*) in parasite isolates from malaria-infected children in Ibadan, Nigeria to evaluate their genetic diversity, relatedness and pattern of molecular evolution. *Pfceltos* and *Pfrh5* genes were amplified from *P. falciparum* positive samples. Amplified fragments were purified and sequenced using the chain termination method. Post-sequence edit of fragments and application of various population genetic analyses was done. We observed a higher number of segregating sites and haplotypes in the *Pfceltos* than in *Pfrh5* gene, the former also presenting higher haplotype (0.942) and nucleotide diversity (*θ* = 0.01219 and *π* = 0.01148). In contrast, a lower haplotype (0.426) and nucleotide diversity (*θ* = 0.00125; *π* = 0.00095) was observed in the *Pfrh5* gene. Neutrality tests do not show deviation from neutral expectations for *Pfceltos*, with the circulation of multiple low frequency haplotypes (Tajima’s *D* = −0.21637; Fu and Li’s *D* = −0.08164; Fu and Li’s *F* = −0.14051). Strong linkage disequilibrium was observed between variable sites, in each of the genes studied. We postulate that the high diversity and circulation of multiple haplotypes has the potential of making a *Pfceltos*-subunit vaccine ineffective, while the low genetic diversity of *Pfrh5* gene substantiates its evolutionary conservation and potential as a malaria vaccine candidate.

## Introduction

Malaria is a substantial public health challenge, especially in sub-Saharan Africa (sSA), where *Plasmodium falciparum* (Pf) is the predominant species. Though much progress has been made in the fight against disease, there appears to be a recent increase in morbidity and mortality, with sSA accounting for 95% of the global number of cases and deaths (WHO, 2021). Nigeria in particular, is responsible for 27% and 31% of global cases and death respectively, demonstrating the unequal impact of disease endured by the continent. Malaria control is fraught with development of resistance to antimalarial drugs and insecticides (Asare et al., 2017; Fadel et al., 2019; Ndiaye et al., 2005; Ngassa-Mbenda & Das, 2016; Opondo et al., 2019; The malERA Refresh Consultative Panel on Tools for Malaria, 2017; Weedall et al., 2019), this becoming more crucial in the face of rising reports of Pf resistance from Uganda (Asua et al., 2021; Balikagala et al., 2021; Fukuda et al., 2021; Rasmussen et al., 2017; Tumwebaze et al., 2021 and Rwanda (Straimer et al., 2022; Uwimana et al., 2020; Van Loon et al., 2022), known to be hotspots for the spread of drug-resistant isolates. There is only one vaccine currently approved for use against malaria, the RTS, S/AS01, with an efficacy less than 50%, which is significantly lower than would have been desired. Another advanced Pf vaccine candidate is the R21/Matrix-M (Osier et al., 2016), which showed a promising outcome two years post-vaccination (Datoo et al., 2022) and has now been adopted by the Nigerian and Ghanaian governments (Erezi, 2023; Grover & Rigby, 2023) for vaccinating children against disease. Both are subunit vaccines, containing a fragment of the *P. falciparum* circumsporozoite protein as the main constituent (Datoo Mehreen S et al., 2021). Despite the benefit of vaccines as disease prevention tools, their effectiveness can be hampered in vaccine escape parasites (Ouattara et al., 2018; Terheggen et al., 2014; Healer et al., 2004), further resulting in reduced potency of the vaccines. Such outcomes could lead to an increase in frequency of resistant alleles in genes mediating parasite escape, and leading to inefficacious vaccines. Thus, this variability of malaria vaccine candidates is a significant drawback hampering the design of efficacious vaccines against disease.

Antigens targeted for vaccine design are expressed at the pre-erythrocytic, erythrocytic or sexual stages (Chauhan, Yazdani & Gaur, 2010; Mahmoudi & Keshavarz, 2018; Ouattara & Laurens, 2015). Both RTS, S/AS01 and R21/MM contain antigens that are expressed at the pre-erythrocytic stage (Datoo Mehreen S et al., 2021). There are additional malaria vaccine candidates, such as the Pf cell-traversal protein for ookinetes and sporozoites (*Pfceltos*), which is involved with ookinete traversal of the mosquito midgut and sporozoites invasion of the human liver cells and has shown promise (Jimah et al., 2016). Others include the *Plasmodium falciparum* reticulocyte binding homologue-like 5 (*Pfrh5*), one of the ligands employed in the invasion of red blood cells by the parasite. It has been shown that if knocked out, the parasite will not be able to invade red blood cells (Patel et al., 2013). Both *Pfceltos* and *Pfrh5* encoded antigens have been reported to be conserved and substantially immunogenic, eliciting significant humoral and cellular response (Bergmann-Leitner et al., 2011; Bergmann-Leitner et al., 2010; Chiu et al., 2014).

The lack of significant polymorphisms across different geographic regions, in addition to their biological importance, supports the screening of these proteins as malaria vaccine candidates. Therefore, we assessed the degree of genetic and haplotype diversity of *Pfceltos* and *Pfrh5* genes in *Plasmodium falciparum* parasites from infected children in Ibadan, Nigeria.

## Materials & Methods

### Ethics

Approval for this study was obtained from the University of Ibadan/University College Hospital Institutional Review Committee (approval number UI/EC/12/0279). The study was conducted in accordance with the Helsinki Declaration of 1975. Written informed consent was obtained from parents or guardians prior to the enrolment of children into the study.

### Study site, sample collection and patient classification

The study site was Ibadan, in Oyo State, Nigeria. Detailed demographic, geographic and clinical parameters, as well as genomic DNA extraction protocol, including modifications, are as described (Ojurongbe et al., 2017). One hundred and twenty-three children presenting with presumptive malaria and for whom informed consent was obtained were included in the study, from November 2013–November 2014. Axillary body temperature and packed cell volume (expressed as a percentage), indicative of fever and anemia respectively were determined, as well as parasite count.

### PCR amplification of *Pfceltos* and *Pfrh5* genes

A 393 bp and 724 bp fragments of *Pfceltos* and *Pfrh5* region of coding sequence were amplified from each sample, using previously designed primers (*Pfceltos* F:5′-CAGAGGAAACAACGGACACA-3′ (nt: 659663-659644) and *Pfceltos* R:5′-TTCGCACCTACAGCTGTTTC-3′ (nt: 659838-659819)); (*Pfrh5* F:5′-CGAAGAATCAAGAAAATAATCTG-3′ (nt: 1081390–1081367) and *Pfrh5* R: 5′-TCTTCGGTTTCATCATCTGT-3′ (nt: 1080685–1080666)) (Oboh et al., 2022; Ochola-Oyier et al., 2016). PCR reactions were carried out using EconoTaq Plus Green 2X Master Mix (Lucigen, Middleton, WI, USA). Each PCR reaction of 25 µl contained: 12.5 µl of the 2X master mix, 5 µl of nuclease free water, 5 µl of gDNA and 1.25 µl of forward and reverse primers (equating to 0.5 mM of each primer). The cycling condition is 95 °C denaturation for 2 min, a second denaturation at 95 °C for 45 s, annealing at 58 °C for 45 s, extension of 72 °C for 1 min for 35 cycles, and a final extension of 72 °C for 5 min. All PCR products were resolved on a 2% agarose gel stained with ethidium bromide, and examined under a BioDocIt.

### Sequencing and sequence analysis

All amplified PCR products were purified using the ExoSAP-IT™ enzyme (Thermo Scientific, Waltham, MA, USA) per the manufacturer’s instruction. Twenty amplicons (selection size due to budgetary constraints) with bright fragments of each target gene were selected randomly, purified and sent to Genewiz-Azenta Life Sciences (South Plainfield, NJ, USA) for bi-directional commercial sequencing using chain termination process. Electropherograms of each sequence was visualized with the BioEdit Sequence Alignment Editor tool (version 7.0.5.3) (Hall, 1999). The 3D7 reference sequences for both *Pfceltos* (Pf3D7_1216600) and *Pfrh5* (Pf3D7_0424100) were downloaded from the NCBI database (https://www.ncbi.nlm.nih.gov) and used to detect single nucleotide polymorphisms (SNPs). Translation of nucleotides into amino acid sequences were carried out and used to determine circulating amino acid haplotypes of each gene at the study site. We utilized various genetic approaches to estimate the haplotype (Nei, 1987) and nucleotide diversities (as estimated by *θ*w and *π*) (Tajima, 1989; Watterson, 1975). The average number of pairwise nucleotide difference per site was estimated by *π* (Tajima, 1989), and the *θ*w value was based on the number of segregating sites in a population (Watterson, 1975). The pattern of immune selection was determined using the Tajima’s *D* test for the two different genes in 100 bp sliding window with a 25 bp step-wise increase. Also, the molecular evolution and linkage disequilibrium (LD) (Barrett et al., 2005) of both genes were determined. DnaSP program version 5.0 (Librado & Rozas, 2009) was used to carry out all evolutionary analyses. In addition, haplotype network was constructed using the Templeton, Crandall and Sing (TCS) model (Clement, Posada & Crandall, 2000) in the PopArt program (Leigh & Bryant, 2015).

## Results

### Description of malaria positivity and amplified gene fragments from Ibadan

Of the one hundred and twenty-three participants included in this study, *P. falciparum* positive samples (56) were subjected to conventional PCR targeting the *Pfceltos* (full CDS length = 549 bp) and *Pfrh5* (full CDS length = 1,578 bp) genes. PCR amplified 44 (35.8%) and 48 (39.0%) for *Pfceltos* and *Pfrh5* genes respectively. Twenty samples of each *Pfceltos* and *Pfrh5* PCR-amplified products were subjected to Sanger sequencing (Table 1). All sequences generated in this study have been submitted to the GenBank (accession numbers: OP484626–OP484662)

**Table 1:** Genetic diversity and pattern of selection of *Pfrh5* and *Pfceltos* from malaria patients in Ibadan, Nigeria.

**Genetic metric**	***Pfrh5***	***Pfceltos***
**Number of isolates**	20	20
**SNPS/Segregating sites**	3	15
**Number of haplotypes**	3	15
**Haplotype diversity**	0.426	0.942
**Nucleotide diversity Θ**	0.00125	0.01219
Π	0.00095	0.01219
**Average number of nucleotide difference**	0.647	4.247
**Tests of neutrality**		
**Tajima’s D**	−0.62614	−0.21637
**Fu and Li’s D**	1.00649	−0.08164
**Fu and Li’s F**	0.64375	−0.14051
**Full length of CDS**	549 bp	1,578 bp
**Length of amplicon studied**	393 bp	724 bp

**Notes.**

*p* > 0.10 for all test of selection (Tajima’s D, Fu and Li’s D/F).

*Pfrh5* *Plasmodium falciparum* reticulocyte binding protein homologue 5 *Pfceltos* *Plasmodium falciparum* cell traversal protein for ookinetes and sporozoites SNP Single nucleotide Polymorphism CDS Coding sequence bp base pair

### Evaluation of genetic diversity, haplotype frequency and network connectivity

Out of the 20 *Pfceltos*-amplified sequencing products, we observed 15 segregating sites with 15 haplotypes. Additionally, a high haplotype diversity (0.942) was observed for this gene while the nucleotide diversity (*θ*w = 0.01219; *π* = 0.01148) was moderate. Furthermore, the average pairwise number of nucleotide difference was 4.247. In contrast, of the 20 sequenced *Pfrh5* isolates, three segregating sites and three haplotypes were observed. Haplotype (0.426) and nucleotide (*θ*w = 0.00125; *π* = 0.00095) diversities were lower than that for the *Pfceltos* gene. The average pairwise nucleotide difference for *Pfrh5* gene is 0.647 (Table 1).

Amino acid mutations at positions 100, 102, 104, 114, 116, 117, 118, 119 and 123 of *Pfceltos* were used to create a 3D7 reference haplotype of KASLSFENE. The most prevalent haplotypes were KASLSVEKE (25%), NALLSFEKE (10%) and RALSVEKE (10%), while others occurred infrequently. With regard to the *Pfrh5* gene, mutations at codon 147, 148 and 203 were used to form only three amino acid haplotypes. The most predominant of these is the YHY (75%), followed by the YHC (15%). The least common haplotype is the HDY(10%) (Table 2).

**Table 2:** Test for pairwise linkage disequilibrium from malaria parasite isolates from Ibadan, Nigeria between *Pfceltos* and *Pfrh5* gene polymorphisms.

**Gene**	**Haplotypes**	**Frequency (%)**
***Pfrh5***	YHC	3 (15)
	YHY	15 (75)
	HDY	2 (10)
***Pfceltos***	KASLSFENE	1
	NALLSFEKQ	1
	KASLSVEKE	5
	NALLSFEKE	2
	KASLPVEKE	1
	KASLSVEKQ	1
	KASLSVEQE	1
	RALSVEKE	2
	RASLSVEKE	1
	KAILSVEKE	1
	RALLSFEKE	1
	RAFLSVEQQ	1
	KAILSVEKA	1
	NASLSFENE	1

**Notes.**

Table displaying the major amino acid haplotypes (three and fourteen observed in the *Pfrh5* and *Pfceltos* gene respectively) and observed in the different isolates. Each letter signifies a particular amino aicd e.g., Y, Tyrosine; H, Histidine; D, Aspartic acid; K, Lysine etc.

Pfrh5 *Plasmodium falciparum* reticulocyte binding protein homologue 5 Pfceltos *Plasmodium falciparum* cell traversal protein for ookinetes and sporozoite

**Figure 1:**
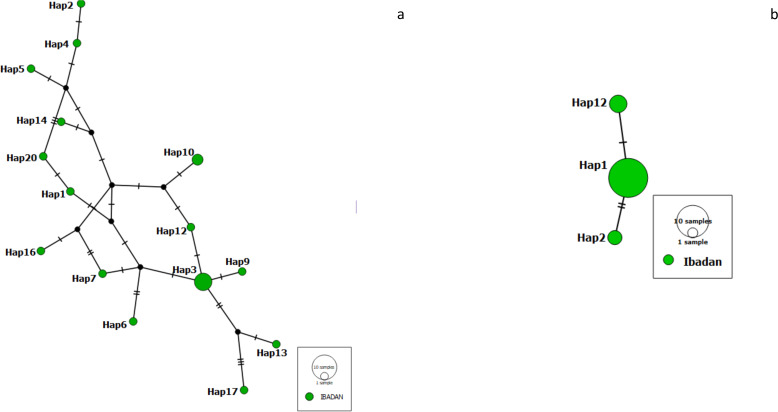
Haplotype network observed in malaria-infected individuals from Ibadan, Nigeria with *Pfceltos* gene (A); and *Pfrh5* gene (B) *Pfceltos* showed multiple haplotypes with low genetic relatedness for *Pfceltos* but not *Pfrh5*. There were only three haplotypes closely related to one another for the *Pfrh5* gene.

We constructed a haplotype distribution map for *Pfrh5* gene by comparing the translated amino acids of the sequence with the 3D7 reference strain at positions 147, 148 and 203 (giving a YHC haplotype), while that of *Pfceltos* gene was carried out by comparing the translated amino acids of the sequence with the 3D7 reference strain at positions 100, 102, 104, 114, 116, 117, 118, 119 and 123 (KASLSFENE). A short haplotype network and reduced variation was observed with the *Pfrh5* gene, while the haplotype network for the *Pfceltos* gene is complex with some haplotypes very distant from each other (Fig. 1).

### Signatures of selection and linkage disequilibrium for *Pfceltos* and *Pfrh5*

The Tajima’s *D* test which calculates the normalized difference between *θ*w and *π* was employed to estimate the signatures of selection. A negative value indicates an excess of low frequency polymorphism which specifies directional selection or population size expansion while a positive value depicts balancing selection or population size reduction. These values in order to validate the direction of selection that they denote are usually confirmed by the Fu and Li’s *F* and *D* test. The Tajima’s *D* value though negative for the *Pfceltos* gene (average of −0.21637) does not deviate from neutral model. Although the average Tajima’s *D* is consistent with purifying selection pattern, the 126–225 and 151–250 base pair sliding window regions showed a positive Tajima’s *D* value of 0.2744 and 0.6981 respectively (Table S1). The Fu and Li’s *D* (−0.08164) and Fu and Li’s *F* (−0.14051) parameters confirmed the observed average purifying selection pattern. Moreover, there was no deviation from the neutral model of evolution (*p* > 0.10).

Further, the Tajima’s *D* value of the *Pfrh5* fragment studied (−0.62614) is consistent without any deviation from the neutral model, while the 375–325, 400–350, and the 425–375 base pair regions have same Tajima’s *D* value (−0.7686) (Table S2) but lower than the Tajima’s *D* of the entire *Pfrh5* gene length. However, this was not confirmed by the Fu and Li’s *D* (1.00649) and *F* (0.64357) tests (Table 1).

**Figure 2:**
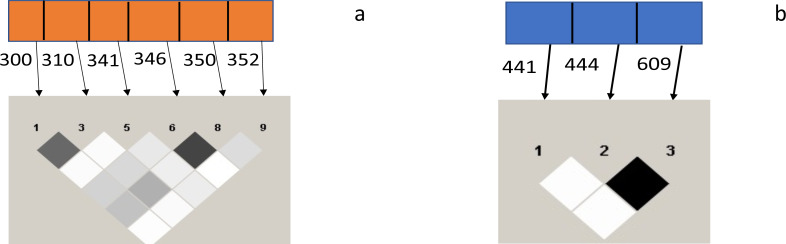
Test for pairwise linkage disequilibrium from malaria parasite isolates from Ibadan, Nigeria between *Pfceltos* and *Pfrh5* gene polymorphisms. The strength of association between pair of SNPs is indicated by the color intensity of the boxes, the darker boxes represent stronger associations, and the lighter ones represent weaker ones. Values within the boxes are the *r*^2^: higher value is indicative of stronger association, and the black boxes shows perfect association (*r*^2^ = 1). (A) *Pfceltos* (chr 12); (B) *Pfrh5* (chr 4).

A strong linkage disequilibrium (LD) was observed between codons 300 and 310 and 346 and 350 of the *Pfceltos* gene, while moderate association was noted between codons 300/346, 310/346, 300/350, and 310/350. With regard to the *Pfrh5* gene, only codon 444 and 609 exhibited a strong linkage disequilibrium (Fig. 2).

## Discussion

One of the hurdles for a potent malaria vaccine is antigenic variation, exhibited by *P. falciparum* at different stages of its life cycle. Pf antigens on the surface of sporozoites, merozoites and other stages, changes the expression on its surface coat to avoid an exposure to the human immune system. This has slowed down the pace of vaccine development. Therefore, while screening for new malaria vaccine candidates, it is imperative to evaluate the diversity and immune selection pattern in various endemic regions.

*Pfceltos* and *Pfrh5* are promising malaria vaccine candidates, which have been shown to trigger strong immune response in infected hosts (Bergmann-Leitner et al., 2013; Bergmann-Leitner et al., 2011; Chiu et al., 2014; Tran et al., 2014). However, due to the paucity of information on the genetic diversity and evolutionary dynamics of these candidates in clinical isolates, particularly from Nigeria, shouldering the heaviest global burden of disease, we set out to elucidate the pattern of evolution and diversity of *Pfceltos* and *Pfrh5* genes from a malaria endemic site in Ibadan Nigeria. Of the total participants enrolled in the study, only about half were positive for *P. falciparum* and ∼79% and ∼86% of these positive isolates were amplifiable for *Pfceltos* and *Pfrh5* genes respectively. The reason for these lower-than-expected rate of gene amplification could be attributed to nucleotides mismatches at the primers annealing sites of these specific isolates.

We detected high number of segregating sites (100, 102, 104, 114, 116, 117, 118, 119 and 123) and haplotypes (15) in *Pfceltos* from our study site. This number of segregating sites is lower than that observed in The Gambia (25), Senegal (26), Mali (20) and French Guiana (22), but higher than what was reported from Iran (nine) and Uganda (14). This might be due to differences in malaria rates between countries plus the degree of endemicity. In addition, differences in experimental design, length of the gene fragment amplified/analysed as the length analysed in the study being compared is longer than the length we amplified in this study, number of sequences analysed—analyses with many sequences as in the case in Iran, Senegal, The Gambia, and Mali while the number of sequences in French Guiana (18) and Uganda (nine) are few compared to what was analysed in our study, and population groups being studied might be contributory factors to the diversity of these segregating sites. Further, the small sample size employed in this study, might also be an added factor responsible for the result observed here. The different polymorphic sites observed in this study (100, 102, 104, 114, 116, 117, 118, 119 and 123) had been observed in Iran, Senegal, The Gambia, Mali, Uganda and French Guiana except amino acid positions 102, 114 and 118 which was observed in our previous study in Nigeria. The uniqueness of these positions (102, 114 and 118) in Nigerian isolates could be due to local parasite adaptation to host immune response. Taken together, these numbers (number of segregating sites) in addition to ours, demonstrate the extensive diversity of *Pfceltos* gene in epidemiologically diverse malaria environments, confirming our hypothesis about its utility as a vaccine candidate. Potentially of more importance is the higher haplotype diversity in Ibadan compared to that reported for The Gambia (0.897) and Mali (0.857) (Pirahmadi et al., 2018). High genetic diversity is an established factor that is known to enhance parasite immune and drug escape (Dutta et al., 2007; Takala & Plowe, 2009). Therefore, the substantial diversity observed in *Pfceltos* will have serious implication on the potency of the *Pfceltos-* subunit or conjugated vaccine.

With regards to *Pfrh5* gene however, the number of segregating sites (three) and haplotypes (three), were fewer as well as haplotype and nucleotide diversity. The number of segregating sites observed from this location is lower than that observed in Lagos, Nigeria (Ajibaye et al., 2020), but comparable to what was observed in Kenya (Ochola-Oyier et al., 2016), while the number of haplotypes is comparable to what was observed in both Lagos and Kenya. The three segregating sites observed show no uniqueness in geographical location as they are present in the malaria endemic areas where *Pfrh5* has been assessed. Furthermore, the haplotype diversity is also comparable to that reported from Lagos, Nigeria (Ajibaye et al., 2020). This significantly reduced nucleotide and haplotype diversities of *Pfrh5* gene and near similar results with isolates from Lagos points to the geographical closeness of both locations and similarity in malaria endemicity. Additionally, the massive back-and-forth movement of goods and individuals on a daily basis provides the opportunity for parasite recombination and premise for similar isolates.

Many haplotypes of *Pfceltos* were circulating in Ibadan but only three (KASLSVEKE, NALLSFEKE and RALSVEKE) were more frequent, while the others had low frequencies. This is a possible indication of the circulation of low frequency alleles that might pose challenge to a *Pfceltos* formulated vaccine efficacy. On the other hand, the most predominant *Pfrh5* haplotype is the YHY, which has been observed in studies from Mali, Burkina Faso and Kenya (Ochola-Oyier et al., 2016; Ouattara et al., 2018) but at a lower frequency. The other haplotypes, YHC and HDY, have also been observed in Kenya (Ochola-Oyier et al., 2016) but none were observed in Lagos, Nigeria (Ajibaye et al., 2020). This inconsistent distribution of the various haplotypes of *Pfrh5* possibly is indicative of the differences in malaria transmission and definable endemicity from different parts of the sub-Saharan African continent. To this end, our results shows some credence to the possibility that a *Pfceltos* conjugated vaccine would fail field trial and prove ineffective. Though these results are unambiguous as to the implication for malaria vaccine design in sub-Saharan Africa, it calls for aggressively focused epidemiological studies in each malaria-endemic locality to document the extent of genetic diversity on disease severity and infection outcome.

The Tajima’s *D* value for *Pfceltos* is consistent with purifying selection but did not deviate from the neutral model. The high number of segregating sites, including high number of haplotypes with low frequencies in the *Pfceltos* gene, is indicative of purifying selection. In spite of the Tajima’s *D* value being indicative of purifying selection, this was not confirmed by the Fu and Li’s test, and did not depart from the neutral model of evolution. The few haplotypes of *Pfrh5* were observed to be closely associated than between the significantly more haplotypes of the *Pfceltos* gene. This, together with the strong linkage disequilibrium observed between codons 300/346, 310/346, 300/350, and 310/350 of the *Pfceltos* gene, may suggest that an interaction exists between single nucleotide polymorphisms. This also has the potential to make a *Pfceltos* subunit vaccine ineffective as it affects the binding affinity of B-cell epitopes, as shown from our previous report (Oboh et al., 2022) and studies of other vaccine conjugates (Bustamante et al., 2013). The negative consequence arising from this has the potential to affect the global effort on malaria elimination by 2030.

## Conclusions

Our study evaluated the genetic diversity and mode of evolution of *Pfceltos* and *Pfrh5* vaccine candidate antigens samples collected from children in a malaria endemic setting in Ibadan, Nigeria and found *Pfceltos* to be more diverse with higher number of segregating sites despite the shorter fragment studied in comparison to *Pfrh5* antigen. The latter, despite the longer fragment length was more conserved and exhibited a very low number of segregating sites. The implication of this is that *Pfrh5* because of its conserved nature has the potential of being a more effective subunit vaccine than the former.

Though there was a limitation on the total number of amplified samples that were sequenced for our analysis, attributable to budgetary constraints, the findings nevertheless present important information that would aid further functional characterization and consideration on how each or combined single nucleotide polymorphisms will impact *Pfceltos* subunit vaccine in the design of next generation malaria vaccines and should be cautiously interpreted. Furthermore, we recommend that the findings mediate much more elaborate and extensive field surveys, with results that would guide future malaria control and implementation programs.

## Supplemental Information

10.7717/peerj.16519/supp-1 Supplemental Information 1 Raw Pfceltos representative sequences

10.7717/peerj.16519/supp-2 Supplemental Information 2 Raw Pf rh5 representative sequences

10.7717/peerj.16519/supp-3 Table S1 Tajima’s D Value in sliding window Pfceltos

10.7717/peerj.16519/supp-4 Table S2 Tajima’s D Value in sliding window Pfrh5

## Additional Information and Declarations

### Data Availability

The following information was supplied regarding data availability: The sequences are available at GenBank: OP484626–OP484662.
